# Pulp from Colored Potatoes (*Solanum tuberosum* L.) as an Ingredient Enriching Dessert Cookies

**DOI:** 10.3390/foods12203735

**Published:** 2023-10-11

**Authors:** Dorota Gumul, Rafał Ziobro, Jarosław Korus, Magdalena Surma

**Affiliations:** 1 Department of Carbohydrate Technology and Cereal Processing, Faculty of Food Technology, University of Agriculture in Krakow, Balicka Str. 122, 30-149 Krakow, Poland; rrziobro@cyf-kr.edu.pl (R.Z.); rrkorus@cyf-kr.edu.pl (J.K.); 2 Department of Plant Products Technology and Nutrition Hygiene, Faculty of Food Technology, University of Agriculture in Krakow, Balicka Str. 122, 30-149 Krakow, Poland; magdalena.surma@urk.edu.pl

**Keywords:** antioxidants, acrylamide, HMF nutritional value, physical features, fortified cookies, pulp from red and purple potatoes

## Abstract

Freeze-dried pulp from colored potatoes, obtained after starch isolation, is a rich source of polyphenols. Therefore, it can be used to fortify cookies, contributing to a reduction in industrial waste, aligning with the zero-waste technology. The purpose of this study was to analyze the effects of adding 5% and 10% pulp from two varieties of colored potatoes on the content of polyphenols, antioxidant activity, physical characteristics, nutritional composition, and the levels of hydroxymethylfurfural and acrylamide of the fortified cookies. The findings revealed that colored potato pulp is an outstanding additive for fortifying cookies with polyphenols, flavonoids, anthocyanins, and flavonols (even two to four times in comparison to control). Cookies containing pulp exhibited even two times higher fiber and protein content (up to 17% more), while the fat and ash content remained unchanged compared to control cookies. Furthermore, they contained 30% less HMF and 40% more acrylamide. These cookies also exhibited good physical properties in the final products. The study demonstrated that pulp from the “Magenta Love” potato variety was significantly more effective in enriching cookies with health-promoting compounds and nutrition value compared to pulp from Marleta Blue.

## 1. Introduction

Processing of raw materials of plant and animal origin contributes to the production of large quantities of organic residues by the food industry. Waste associated with the processing of meat and animal products accounts for 13%, while losses associated with the processing of fruits and vegetables are 22% [1]. These are both solid and liquid materials, which usually have no further use in the production chain. If not properly treated, they can increase the pollution of soil, surface water, and groundwater [2,3,4,5].

Starch manufacture is one of the processing methods that involves the occurrence of large quantities of by-products, which may be dangerous for environment. Three types of organic waste are generated during the production of potato starch: potato pulp, potato juice, and juicy water. For every ton of starch produced, there are 1.36 tons of potato pulp, 0.14 cubic meters of potato juice, and 3.66 tons of juice water [6]. Potato juice is a fluid separated from potato pulp containing about 5% dry mass including potato protein of high nutritional value. Potato juice contains 2% nitrogenous compounds, which include protease inhibitors, lectins, phosphorylases, and kinases. The juicy water is produced during the refining of starch milk. It is a tenfold dilution of potato juice. Due to its high content of water and low caloric value, the incineration of solid starch waste does not seem rational. Potato pulp is a by-product obtained after washing out the starch from the disintegrated potato cells with water. The pulp consists mainly of bound starch inside intact cells, minerals (Ca, Cl, Na, P, Mg), and insoluble non-starchy substances including fiber, of which polyphenols are an integral part. Solanine and chaconine, which are toxic glycoalkaloids present in raw potatoes, make up only 0.006% of the potato’s fresh weight and are water-soluble and leached out with water during this processing process [7].

Annual production of potato pulp in Europe exceeds one million tons. In northern Japan, about 100,000 tons are produced every year. The pulp is mainly used as fodder or fertilizer. However, these methods are not economical. Still a large part of the waste remains unmanaged, which affects the additional disposal costs incurred by the plant and increases the biological load of wastewater [8]. Potato pulp consists primarily of cell wall fragments containing residual starch. It contains up to 30% pectins, which could be used in the production of jellies and jams [9]. Pectins are water-soluble esters of polygalacturonic acid, partially esterified with methyl alcohol. They are formed from water-insoluble protopectin. In combination with acids and sugar, they may produce stiff gels. We can distinguish between high-methylated and low-methylated pectins [9], which are used for the confectionery and pastry industry and in the production of jellies and jams and beverages. Fermentation of potato pulp could be applied for the production of butanol or acetone. Some attempts have also been made at enzymatic hydrolysis of potato pulp in order to obtain protein preparations [10].

Considering the possible applications of potato pulp, no attention has been paid to polyphenols, which are an integral part of dietary fiber contained in the pulp. Their presence in significant quantities gives a possibility to obtain food products, such as cookies, with improved nutritional and health-promoting properties, maintaining the attractiveness of the final product.

The production of confectionery products is an important branch of the food industry, as the consumption of sponge cakes, cookies, and other confectionery products continues to grow. The global confectionery market is projected to reach $270.5 billion by 2027 (Allied Market Research Report) [11]. Although nutritionists underline that cookies are high in fat and sugar and could be partly responsible for obesity, manufacturers are trying to change this negative perception of confectionery products by introducing health-promoting, nutrient-enriched, reduced-calorie raw materials, or eliminating sugar or replacing it with other sweeteners, and supplying vitamins, fiber, and polyphenols. Efforts are also being made to introduce new products for people with celiac disease or gluten intolerance, who due to the elimination diet could suffer significant oxidative stress and impaired antioxidant enzymes, which normally form a specific antioxidant barrier in the body. Such people are susceptible to oxidant–antioxidant imbalance and DNA damage, and consequently to cancer [12,13], which may partly be prevented by introducing variability of different foods. Such gluten-free products will unquestionably include the cookies with colored potato pulp that are the subject of this manuscript’s research.

It should be added that cookies have become a commercially important product due to the practicality of their production and the long shelf life of the finished products. However, customers are looking for new innovative products as the market develops rapidly. The average appearance and quality of the raw materials from which the product is made no longer satisfy the consumers. They want new products with surprising attractive properties and nutritional value, and above all with health-promoting properties. Therefore, producers are looking for new solutions, positively influencing the foodstuffs, preferring not only the pro-health values of products, but also paying attention to harmful substances (acrylamide and HMF), which are formed as products of the Maillard reaction during baking.

The possibilities of enriching cookies with raw materials of high antioxidant potential can be directed towards the introduction of new products on the market, especially for celiacs. On the one hand, the possibility of utilization of the waste product, such as potato pulp, and on the other, the growing awareness of consumers, who look for chemopreventive food ingredients, make it possible to introduce new products on the market. Therefore, this research fits into the current global trend of functional food [14,15,16,17,18,19].

The aim of this study was to analyze the effect of the level (5 and 10%), origin (two colored flesh cultivars) of potato pulp on the content of polyphenols, phenolic acids, anthocyanins and flavonoids, and the antiradical activity (using the free radical ABTS) in dessert cookies. In addition, the study focused on the physical characteristics of these cookies and their nutritional value. Furthermore, acrylamide and hydroxymethylfurfural (HMF) were determined in the final product.

## 2. Materials and Methods

### 2.1. Pulp Extraction and Preparation of Cookies Enriched with Potato Pulp

The batch of potatoes was washed under running water, then a certain part of it was mashed into a homogeneous pulp and transferred in portions to a mill gauze placed on a Buechner funnel, and the starch was washed by pressing the gauze by hand. The leaching process was stopped when the filtrate collected from under the sieve showed a negative reaction with Lugol’s iodine. A negative color reaction with iodine allowed the next portion of the pulp to be taken for starch isolation. The pulp was immediately placed in a freezer for about 1 week, 1 day of blast freezing, and freeze-dried. The efficiency of the potato pulp extraction process was 50%. The subjects of the study were dessert cookies (DC) baked under laboratory conditions from corn and potato starches enriched with 5 and 10% of freeze-dried potato pulp. The potato pulp was made from colored flesh potatoes of two varieties, Magenta Love (ML) (red potatoes) and Marleta Blue (MB) (purple potatoes). Potato pulp was obtained as a by-product in laboratory isolation of potato starch, as described above, according to Wischmann et al. [20], subsequently freeze-dried, and included in cookie formulations.

The dough consisted of the ingredients listed in Table 1. The recipe for dessert cookies included corn starch (Bezgluten, Posądza, Poland) and potato starch (Pepees SA, Łomża, Poland). The ingredients, after being weighed according to the recipe (Table 1), were mixed in a Diosna spiral mixer type SP 12 (Osnabrück, Germany) for about 5 min at slow speed until the dough was kneaded. The dough was then rolled out using a confectioners’ rolling pin to obtain an even thickness. The cookies were punched out using a single mold with a diameter of 5 cm. Baking was carried out at 230 °C for 12 min. A MIWE CO 2 P608 type oven (Arnstein, Germany) was used for baking. After the cookies cooled to room temperature, they were packed into glass containers (jars) and stored in a storage chamber at a constant temperature of 22 °C, for further analysis.

### 2.2. Examination of the Physical Characteristics of Cookies Enriched with Potato Pulp

To measure the physical characteristics, 6 pieces of cookie were weighed with an accuracy of 0.1 g, and the average weight of one cookie was calculated. The length of the segment occupied by each cookie was measured in mm, the measurement was repeated in perpendicular axis, and the average diameter of one cookie was calculated. The thickness of the cookies was measured with a ruler by placing all the cookies one on top of the other. The spread ratio was calculated according to the following formula:

S = W/T

where S—spread ratio, W—diameter of 1 cookie (mm), T—thickness of 1 cookie (mm). Volume of cookies was determined using Volscan 600 (Stable Micro Systems, Surrey, UK) (Supplementary Materials Figure S1) placing all cookies on the rotating base and dividing the result by the number of stacked cookies.

### 2.3. Chemical Evaluation

The following analyses were performed on each sample:

1. Chemical composition—content of protein, fat, ash—was determined according to AOAC 2006 [21]. Total protein content was carried out using Kjeltec (2200, FOSS, Denmark), according to the AOAC method No 205,950.36. Evaluation of the raw fat content was carried out by using Soxtec Avanti extractor (2055, FOSS, Denmark), following the AOAC method No. 930.05. Total ash content was carried out by using laboratory stove model SM-2002 (Czylok, Poland), following the AOAC method No. 930.05. Available carbohydrates were calculated by difference: 100—fat-protein-ash-water-dietary fiber. Energy value of the cookies was calculated according to EU Directive 1169/2011 [22]: available carbohydrates 4 kcal/g, protein 4 kcal/g, fat 9 kcal/g, fiber 2 kcal/g.
2. Content of non-starch polysaccharides, i.e., total, dietary fiber, by the method 32-07 of AACC [23].
3. Antioxidant constituents, antiradical activity was determined in the ethanol extracts. A total of 0.6 g of the sample was dissolved in 30 mL 80 g/100 g ethanol, shaken in darkness for 120 min (electric shaker: type WB22, Memmert, Schwabach, Germany), and centrifuged (15 min, 4500 rpm. 1050× *g*) in a centrifuge (type MPW-350, MPW MED. Instruments, Warsaw, Poland). The supernatant was decanted and stored at −20 °C for further analyses. Determination of total polyphenols content (TPC) was carried out by spectrophotometric method using Folin–Ciocalteu reagent (with F-C reagent), according to Singleton, Orthofer, and Lamuela-Raventós [24], and the content of flavonoids was evaluated using a spectrophotometrical method, according to El Hariri, Sallé, and Andary [25]. Contents of total phenolic content without F-C reagent, phenolic acids, flavonols, and anthocyanins were evaluated using a spectrophotometrical method, according to Mazza et al. [26] and Oomah et al. [27]. Additionally, antiradical activity was assessed using analytical methods with ABTS (2,2′-azino-bis (3-ethylobenzothiazoline-6-sulphonic acid)-diamonium salt) [28]. Results of antiradical activity were expressed as TEAC (Trolox Equivalent Antioxidant Capacity-mM Trolox/kg dry mass of sample).
4. The content of acrylamide (AA) has been determined. For sample preparation according to Surma, Sadowska-Rociek, Cieślik, and Sznajder-Katarzyńska [29] modified QuEChERS method was applied. AA qualitative and quantitative analysis were performed using HPLC-UV/Vis (according to methods [30]. AA determination was performed using the HPLC-DAD system (VWR HITACHI, LaChrom ELITE, Merck KGaA, Darmstadt, Germany) according to Marconi et al. [30]. The chromatographic separation was performed at room temperature using a mixture of 0.01 M sulfuric acid and water/methanol mixture (water:methanol, 97.5:2.5) in isocratic mode with a C18 reversed-phase column (Lichrospher^®®^ 100, RP-18 end-capped, LiChroCART^®®^ 4 mm ID × 250 mm, 10 µm, Merck KGaA, Darmstadt, Germany). The flow rate was 0.7 mL min^−1^. AA was determined at 200 nm wavelength. The identification of acrylamide was based on the retention time (4.2 min). The measurements were performed in four replications.
5. In order to determine hydroxymethylfurfural (HMF) content, 10 mL of water was added to 2 g of samples and quantitatively transferred to a 25 mL volumetric flask. After adding 0.25 mL of Carrez solution I and 0.25 mL of Carrez solution II, the volumetric flask was filled to the mark with deionized water. In the case of infusions, 0.07 mL of Carrez solution I and 0.07 mL of Carrez solution II were added to 6.86 mL of the infusion. Before a chromatographic analysis, samples were filtered through a 0.45 µm disc filter. Reagent blank samples were prepared according to the appropriate procedure for all tested analytes. Each sample (real and blank) was prepared in triplicate.
6. HMF qualitative and quantitative analyses were performed using HPLC-UV/Vis system (VWR HITACHI, LaChrom ELITE, Merck KGaA, Darmstadt, Germany) operating under the following conditions: sample volume 20 µL, eluent water/methanol 9:1 (*v*/*v*), flow rate 1 mL/min, wavelength 285 nm, column RP-18 Lichrosphere (250 × 4 mm, 5 µm particle size) (Merck, Germany).

#### Statistical Analysis

To assess the significance of differences between the averages, the experimental data were subjected to one-way analysis of variance (Duncan’s post hoc test), at the confidence level of 0.05, by the use of software Statistica v. 8.0 (Statsoft, Inc., Tulsa, OK, USA). The Pearson correlation coefficients between selected parameters were also calculated to establish the relationships between them.

## 3. Results and Discussion

### 3.1. Characteristics of Pulp Derived from Colored Potatoes

The content of different subgroups of polyphenols in the potato pulp is shown in Table 2. It was found that the amount of polyphenols determined with Folin-Ciocalteu reagent was about 80% higher in the pulp derived from Magenta Love as compared to Marleta Blue. Similarly, the amount of polyphenols determined by the method without the Folin-Ciocalteu reagent was 86% higher for Magenta Love potato in comparison to Marleta Blue. Also, the potato pulp derived from variety Magenta Love had 31% more flavonoids than Marleta Blue. In the case of phenolic acids, Magenta Love potato pulp had two times higher content of these components than Marleta Blue samples (Table 2). Phenolic acids are among the most numerous phenolic compounds in the potato tuber. The dominant one is chlorogenic acid and its two isomers: neochlorogenic and cryptochlorogenic. Phenolic acids are referred to as nutraceuticals, i.e., enrichment substances with health-promoting effects [31,32,33]. They belong to biologically active compounds that influence the course of many biological processes such as adaptive or repair activities of the system into which they are introduced with food. On the other hand, Marleta Blue’s potato pulp had six times higher content of flavonols than Magenta Love (Table 2). Taking into account the aforementioned pulps, it was found that the one derived from Marleta Blue potatoes contained 52% less anthocyanins than Magenta Love. Overall, the high content of all subgroups of polyphenols in Magenta Love’s potato pulp translated into a high antioxidant potential of this plant material (Table 2).

Taking into account the chemical composition, the analyzed samples did not contain fat, and the product from the red potato variety Magenta Love was characterized by higher protein, ash, and total fiber contents by 48%; 18%, and 17.7%, respectively, in comparison to the variety Marleta Blue (Table 2).

Summarizing, it has been generally demonstrated that colored potato pulp is a source of health-promoting compounds (polyphenols). Such compounds exhibit hypoglycemic, hypocholesterolemic, anticancerogenic effects, and reduce postprandial glycemia and hypertension. They have anti-inflammatory, antiviral, antibacterial, anti-allergenic, and anti-coagulant effects and reduce the risk of diseases such as atherosclerosis and other cardiovascular diseases, cataracts, diabetes, genetic damage, degenerative bone changes, neurodegenerative diseases including Alzheimer’s disease [34,35,36,37,38,39]. Therefore, colored potato residue can be used as a health-promoting ingredient for cookies.

### 3.2. Effect of Colored Potato Pulp on the Content of Health-Promoting Compounds in Cookies

The content of different groups of polyphenols in cookies with the participation of colored potato pulp is presented in Table 3. The amount of polyphenols in control cookies was at the level of 22.07 mg of catechin/100 g d. m. This should most probably be explained by the presence of Maillard reaction products, which affect the results of this determination, because according to other authors [40,41] the Folin-Ciocalteu reagent used in this determination not only reacts with polyphenols but also with other compounds such as amino acids, proteins, saccharides, i.e., compounds that can form Maillard reaction products.

The presence of polyphenols was also noted in control gluten-free bread in studies by other authors [42,43], which was also explained by the production of Maillard compounds. In the current study, the amount of Maillard compounds is much higher, because they are formed via the exposure of the cookies to high oven temperatures. It was observed that the introduction of colored potato pulp in the cookies resulted in an increase in polyphenols ranging from 38 to 115% compared to the control. The smallest increase in polyphenols in the cookies was observed when 5 and 10% of Marleta Blue potato pulp and 5% of Magenta Love potato pulp were used, and the largest when 10% of Magenta Love preparation was added. Such a large increase results from the fact that the amount of polyphenols in the Magenta Love pulp was significantly higher than in the Marleta Blue (Table 2 and Table 3). The determination of polyphenols without Folin–Ciocalteu reagent was also used in this work, giving smaller and more meaningful values compared to those obtained with this reagent. It was noted that the Marleta Blue potato pulp did not contribute to an increase in polyphenols in the cookies regardless of its proportion in the formulation (5 and 10%). Although 5% addition of Magenta Love pulp had no significant influence on polyphenols, its 10% addition caused a 2.5-fold increase in polyphenols in the cookies compared to the control (Table 3). Sudha et al. [44], in a study on the quality of cakes with apple pomace, reported an increase in total polyphenol content from 42.7% to 68.4% in the final products compared to the control. According to the above-mentioned authors, this was related, on the one hand, to the use of apple pomace, from which the polyphenols were derived, and, on the other hand, to the possibility of the formation of products such as reductones. These compounds are formed during the baking process by the oxidation of sugars derived from apple pomace [44]. The addition of 5 to 20% mango peel powder to a cookie recipe resulted in an 8.5-fold increase in polyphenol content [45]. Bertagnolli et al. [46] developed a cookie recipe using flour obtained from guava peels at 30, 50, and 70% substitution level, which resulted in an increase in the polyphenol content of the final product. Kopeć et al. [47] conducted a study on the replacement of wheat flour in sponge cake and shortcrust pastries by buckwheat flour, maize flour, and a mixture of these flours in a ratio of 1:1. The sponge cake and pastries with buckwheat flour had the highest polyphenol content compared to control products containing only wheat flour. In cookies containing dietary fiber isolated from Doum fruit (*Hyphaene thebaica*), the content of polyphenols increased with increasing fiber content. The highest content of these components was determined in cookies with 10% of isolated fiber derived from Doum fruit [48].

The flavonoid content of cookies containing colored potato pulp was 23% higher when 5% Marleta Blue potato pulp was used and four times higher when 10% Magenta Love potato pulp was used, relative to the control (Table 3), with the amount of these health-promoting components increasing accordingly to the level of addition of different types of potato residue. Korus et al. [17] conducted a study on gluten-free cookies using 20%, 40%, and 60% acorn seed flour and hemp flour. Based on the results, it was found that as the amount of hemp and acorn flours increased in the cookies, the flavonoid content increased. The increase in flavonoid content in cookies with acorn flour was five-fold and in cookies with hemp flour 90% compared to control cookies. Maner et al. [49] developed a recipe for cookies enriched with 5, 10, 15, and 20% addition of dried grape pomace. They found that the addition of pomace increased the flavonoid content from 96 to 254% in the final products compared to the control baked goods. Ajibola et al. [50] prepared cookies using dried Moringa oleifera leaves and cocoa powder. They found that 10% participation of Moringa oleifera in the cookie recipe increased the flavonoid content from 0.22 mg/g in the control product to 1.27 mg/g in the final product. Moringa has significant nutritional and health-promoting values, but some studies indicate serious side effects caused by its consumption, so its use in food is not allowed in some countries [51]. Natukunda et al. [52] made a cookie recipe with 2, 4, 6, 8, and 10% tamarind seed powder. The addition increased the flavonoid content from 32 to 153% compared to the control. An increase in flavonoid content as a result of the proportion of purple rice flour was also found by Klunklin and Savage [53]. In the prepared cookies, the flavonoid content was three to eight times higher compared to the control. Van Toan and Vu Quynh Anh [54] used a proportion of 10 to 50% purple sweet potato flour in a cookie recipe. The cookies had a flavonoid content ranging from 5.25 mg quercetin/g to 7.51 mg quercetin/g. The addition of sweet potato flour increased the flavonoid content compared to the control sample of 4.8 mg quercetin/g. When cookies were enriched with acorn flour, Pasqualone and co-workers [55] showed a seven-fold increase in flavonoid content with 30% and a 21-fold increase in the mentioned component with 60% acorn flour addition compared to the control sample.

Dessert cookies with the addition of Marleta Blue pulp contained 36% less phenolic acids at 5% and 32% less phenolic acids at 10% potato pulp compared to control cookies. Also, a 26% decrease in phenolic acids was observed with 5% of Magenta Love potato pulp compared to the unenriched products. It was found that only a 10% addition of Magenta Love preparation caused a two-fold increase in phenolic acids content (Table 3). Analyzing the results, it can be concluded that the low content of phenolic acids in the cookies with Marleta Blue potato pulp at both 5 and 10% addition level may be due to the content of these components in the pulp being lower by half compared to Magenta Love pulp (Table 2 and Table 3). The results were significantly influenced by the baking process of the cookies, which is associated with the decarboxylation of phenolic acids. An opposite effect was obtained by enriching the cookies with 60% acorn flour and hemp flour, which caused an approximately six-fold increase in phenolic acids content in the cookies compared to the control product [17].

Cookies with 5 and 10% share of the potato pulp isolated from variety Marleta Blue contained about 174% more anthocyanins compared to the control cookies. The highest anthocyanin content among was found in cookies with 10% of Magenta Love potato pulp (Table 3). Anthocyanins are characterized by thermal instability. The mechanism of anthocyanin degradation is not fully understood, and research suggests that first the glycosidic bonds in the pigment molecule are hydrolyzed and then an unstable aglycone is formed. Elevated temperature also affects the transformation of anthocyanins into colorless chalcones, which undergo oxidation to form high molecular weight brown compounds and dyes. The degree of anthocyanin degradation during thermal treatment depends on the time and temperature of the process. A logarithmic relationship was observed between the destruction of anthocyanins and heating time at constant temperature. The loss of these components also depends on other factors such as pH, the chemical structure of anthocyanins, and the presence in their environment of oxygen, other polyphenolic compounds, proteins, sugars, and their degradation products. Greater glycosidation of anthocyanins and acylation with phenolic acids improves resistance to high temperature [56]. Maner et al. [49] prepared cookies in which part of the wheat flour was replaced by the addition of dried grape pomace. The addition of pomace affected the dark brown color of the products. The color change was influenced by anthocyanins present in grape skin (mainly malvidin 3-O-glucoside and peonidin 3-O-glucoside). The above-mentioned authors found a significant increase in the anthocyanin content (3.512 mg/g) in cookies with pomace addition compared to the control (0.163 mg/g). In a study by Korus et al. [17], the anthocyanin content of cookies with 60% acorn flour was significantly higher than the control cookies. Also in the same study, it was found that cookies with 60% hemp flour showed 75% higher anthocyanin content compared to cookies containing corn flour. Klunklin and Savage [53] made cookies containing between 25 and 100% purple rice flour in their formulation. With the addition of purple rice, a significant increase in anthocyanin content was demonstrated. Wheat cookies (control sample) contained 0.28 mg/kg d.m. of anthocyanins while cookies baked exclusively with rice flour had a content of 51.49 mg/kg d.m.

An increase in total polyphenols and their subgroups resulted in an increase in antioxidant activity in cookies with red potato pulp (Table 3) relative to the control as evidenced by the strong correlation between TPC and ABTS R = 0.921. High antioxidant activity was observed in cookies with 10% Magenta Love potato pulp (38.15 m M Tx/kg d.m.) relative to the control sample (15.78 mM Tx/kg d.m.). An increase in antioxidant activity was also shown in cookies containing grape pomace [49]. The control cookies had an activity of 4.625 mg Tx/g, while the antioxidant activity of the enriched products was 11.651, 29.669, 51.862, and 75.976 (mg Tx/g) at 5, 10, 15, and 20% pomace content, respectively [49]. Pasqualone and co-workers [55] observed an almost 40-fold increase in antioxidant activity in cookies with acorn flour at 60% compared to control cookies. Klunklin and Savage [53] replacing wheat flour with purple rice flour observed an increase in ABTS radical scavenging capacity from 5.49 μmol Tx/g d.m of control cookies to 95.96 μmol Tx/g d.m when wheat flour was completely replaced by purple rice in the final product. In cookies enriched with dietary fiber from Doum fruit, an increase in antioxidant activity was also observed from 2.65 (mM Tx/100 g d.m.) in the control sample to a content of 18.34 (mM Tx/100 g d.m.) with 10% of the said fiber. The increasing antioxidant activity was shown to be adequate for the polyphenol content of the cookies [48].

### 3.3. Chemical Composition of Cookies with Colored Potato Pulp

Considering the protein content, it was found that the 5% addition of Magenta Love potato pulp did not cause changes in this component compared to the control. The share of 5 and 10% potato pulp isolated from variety Marleta Blue contributed to a small but significant decrease in protein relative to the control. A higher amount of protein was recorded in cookies with a share of 10% potato pulp isolated from variety Magenta Love (Table 4). No fat was determined in the samples, and the amount of ash was identical in each sample analyzed (Table 4). The share of 5% potato pulp isolated from variety Marleta Blue contributed to a two-fold decrease in dietary fiber in relation to the control. The 10% share of potato pulp isolated from variety Marleta Blue contributed to a 60% increase in fiber, and the 10% share of potato pulp isolated from variety Magenta Love contributed to a two-fold increase in dietary fiber in relation to the control (Table 4).

Considering the available carbohydrates, it was found that the share of pulp from colored potatoes reduced their amount in dessert cookies (in the case of a 10% share of pulp by an average of 3.5%) in relation to the control, also decreasing their energy value in relation to the standard (Table 4).

### 3.4. Acrylamide and HMF Content in Cookies with Colored Potato Pulp

No differences were noticed in the HMF contents present in the cookies (Table 5). The only statistically significant difference can be seen when comparing the HMF content of the control sample with the other variants regardless of the percentage of pulp and the varieties of potatoes used in the experiment. In each of the variants tested, the HMF content decreased from 32 to 37% (or about 35%) compared to the control sample (Table 5).

In the case of acrylamide, each addition of pulp regardless of its percentage of the variety of potatoes used resulted in a statistically significant increase in its content relative to the control sample. In the case of the Marleta Blue variety, a 5% addition of potato pulp caused a 22% increase in acrylamide content, while a 10% addition caused a 38% increase in acrylamide content relative to the control sample. On the other hand, the addition of Magenta Love potato pulp caused a 37 and 47% increase in acrylamide content for the 5 and 10% additions, respectively. All the results obtained are statistically significantly different from each other if we consider the percentage of the addition of the pulp. Comparing between each other cookies with the same content of 5 and 10% pulp, respectively, higher acrylamide contents were recorded for the Magenta Love variety, by 12 and 11%, respectively (Table 5), which would suggest that the mentioned variety is characterized by a higher content of amino acids (especially asparagine) or reducing sugars. Although there are no limits for acrylamide in food, according to Commission Regulation (EU) 2017/2158 [57], benchmark levels for the presence of acrylamide in foodstuffs have been established. Food was classified in 10 categories, one of which are cookies and wafers for which the benchmark level for the presence of acrylamide has been set at 350 μg/kg. Taking into account the content of acrylamide in the studied samples with or without potato pulp, the detected levels of this component were lower than benchmark level.

In conclusion, it is possible to propose the practical use of pulp from red and purple potatoes for the production of dessert cookies, especially for people with celiac disease. It should be noted that allergies and food intolerances, including celiac disease, according to recent estimates, are the third major threat to human health right after cancer and cardiovascular disease. People with gluten intolerance are forced to exclude products containing gluten, such as most cereal products, from their diet, which can result in deficiencies of many substances valuable for health, such as protein, vitamins (folic acid, B vitamins), minerals (Fe, Ca, Mg, Cu), and dietary fiber [13,58]. Restriction of the above-mentioned components, in turn, induces many diseases in people with celiac disease, such as osteoporosis, esophageal cancer, or infertility, etc. [59]. In addition, recent studies indicate that patients with gluten intolerance have been determined to have significant oxidative stress and impaired antioxidant enzymes, susceptibility to DNA damage and, consequently, cancer [12]. Proposed cakes involving potato pulp were characterized by up to two times higher content of polyphenols and phenolic acids, four times higher content of flavonoids and anthocyanins, two times higher amount of fiber, and 17% higher amount of protein. Another important practical aspect of the use of red and purple potato pulp to enrich dessert cookies with health-promoting compounds such as polyphenols is that these products can be suggested as an alternative for the treatment of type 2 diabetes, since according to Sun and Miao [60] polyphenols have an inhibitory effect on the digestion of starch, and consequently reduce the glycemic index of starchy products. This is because polyphenols inhibit the activity of the amylolytic enzymes (alpha-amylase and gamma-glucosidase) as well as interact directly with starch, forming hardly digestible complexes. The alleviation of postprandial hyperglycemia by polyphenol compounds might be due to both the inhibited starch digestion in vivo and the influenced glucose transport [60].

### 3.5. Influence of Colored Potato Pulp on Physical Characteristics of Cookies

Table 6 shows the physical characteristics of the dessert cookies with potato pulp isolated from the colored flesh varieties Magenta Love and Marleta Blue. The addition of potato pulp to the traditional cookie recipe resulted in a slight increase in the volume of the final products. In terms of weight, there was little difference from the control products, with the exception of the cookies with 10% of Magenta Love pulp, whose weight was significantly higher than the other samples (Table 6). In the study by Van Toan and Vu Quynh Anh [54] on cookies with colored sweet potato flours, it was found that as the sweet potato flour content increases, the volume of the cookies decreases. This effect may be influenced by the fiber present in the sweet potato flour, which reduces the gas retention capacity of the dough [61,62]. Another factor will be the form of the enrichment additive. It should be noted that the volume of the product is influenced by the form of the additive (whether it is ground or not). The unground form actually breaks the gluten matrix, resulting in a lower volume of the final product [63]. In the case of the study performed in this work, the red potato pulp is ground, and therefore we may be dealing here with an increase in cookie volume (Table 6). Yadav et al. [64] showed a decrease in cookie weight with the addition of banana flour and chickpea flour. The weight of cookies ranged from 8.7 g to 10.1 g with a control cookie weight of 11.4 g. Cookies containing mango seed flour had a 1 to 6% lower weight compared to the control cookies [65]. An increase in the weight of the products was noted for cookies containing rice bran, broken rice, and okara [66]. A similar effect was noted with the contribution of carrot and beetroot powder in the cookie recipe [67]. Srivastava and Singh [68] prepared cookies with 5, 10, and 15% beetroot powder. Their study found no significant differences between the control cookie and the enriched products, and the same was true in the study of this work.

In addition, it was shown that with an increase in the proportion of pulp, the diameter of the cookies decreased in a range of 6.5 to 4% for the Marleta Blue potato variety and 4 to 8% for the Magenta Love cookies compared to the control. The thickness of the cookies increased while the value of the spread ratio decreased as a result of the addition of potato pulp in the final products (Table 6). The spread ratio (width divided by thickness) is considered one of the most important quality parameters for cookies as it correlates with the texture and consistency of the cookie. It is easy to see that the addition of different amounts of flours affects the quality of confectionery [69]. Van Toan and Vu Quynh Anh [54] noted that an increase in the proportion of purple sweet potato flour increased the spread ratio for cookies enriched with the aforementioned flour. The results were directly influenced by the thickness of the cookies decreasing with the addition of purple sweet potato flour, which is directly related to the spread ratio of the products. The diameter value was virtually unchanged regardless of the proportion of sweet potato flour. As a result, cookies made from composite flour had a higher spread ratio than wheat cookies. The result may have been influenced by the lower protein content of the purple potato flour. Makinde and Taibat [70] prepared a cookie recipe with the following flours: maize, almond, and coconut. The contribution of the flours increased the diameter of the cookies from 4 to 8% compared to the control sample of 49.60 mm. The thickness, on the other hand, decreased from approximately 10 to 22% compared to cookies containing only wheat flour. In the final products, the value of the spread ratio increased from 5 to 26% from that characterizing the control cookie. Adeola and Ohizua [71] showed that as sweet potato flour and banana flour increased in the composition of the cookies, the thickness of the cookies increased. These changes were explained by the increasing protein content of the product. An analogous trend was observed in the study of this work because a greater thickness of cookies corresponded to higher protein content (Table 4 and Table 6).

## 4. Conclusions

1. It was found that the Magenta Love potato pulp had a higher content of polyphenols, flavonoids, phenolic acids, and anthocyanins compared to the Marleta Blue variety of potato pulp, which contributed to higher antioxidant activity.
2. It was noted that the Magenta Love potato pulp added at a level of 10% contributed to a significant increase in the total polyphenol content of the dessert cookies compared to the control sample. In addition, an increase in flavonoid content was observed as a result of the use of colored potato pulp in the cookie recipe, particularly when using the potato variety Magenta Love. It was found that the 10% share of Magenta Love potato pulp had a doubling effect on the phenolic acid content of the final product compared to the control cookies. In other cases, a decrease in phenolic acid content was observed, which could be due to degradation processes of these compounds. It was found that the antioxidant activity of dessert cookies with Magenta Love and Marleta Blue potato pulp was higher in relation to the control cookies, which is adequate to the polyphenol content.
3. Considering nutrients, cookies with 5 and 10% Magenta Love potato pulp had higher amounts of protein and fiber compared to the control and cookies with Marleta Blue potato pulp. Cookies enriched with potato pulp had a 30% lower HMF content relative to the control and a higher acrylamide content, which still fell below the limit for this type of product.
4. It was observed that the addition of pulp isolated from colored potatoes increased the volume of the final products relative to the control products. In contrast, the diameter of the analyzed cookies decreased with an increase in the addition of pulp.
5. In conclusion, the pulp isolated from Magenta Love variety was more beneficial for enriching dessert cookies with health-promoting compounds than the Marleta Blue preparation. It can be suggested that dessert cookies with a 10% share of potato pulp of the Magenta Love potato variety can be recommended on an industrial scale because of the product’s health-promoting properties and good physical and nutritional quality. The utilization of by-products in food technology is very important as it is inextricably linked with zero-waste technology. It should be remembered that starch is produced from light-colored potato varieties, which are poorer in phenolic compounds than potatoes with colored flesh. In the future, however, it would be possible to use potatoes with colored flesh for starch production because they are more resistant to diseases due to the very high proportion of anthocyanins in their tubers. Thus, it would be advantageous to use colored potato pulp for the production of food products, especially those labelled gluten free, as it could provide additional value. The gluten-free food sector could be one of the most profitable food industries, not only because of the significant number of people with celiac disease, but also because of the prevailing trend where a significant number of people are switching to a gluten-free diet in search of diets alternative to the traditional one.

## Figures and Tables

**Table 1:** Recipes for dessert cookies with the addition of potato pulp (g).

	Control	DCMB5	DCMB10	DCML5	DCML10
Corn starch	358	340.1	322.2	340.1	322.2
Potato starch	16	16	16	16	16
Icing sugar	180	180	180	180	180
Margarine	96	96	96	96	96
Egg mass	26	26	26	26	26
Milk	64	64	64	64	64
Salt	1.2	1.2	1.2	1.2	1.2
Baking powder	12.8	12.8	12.8	12.8	12.8
Potato Pulp	0	17.9	35.8	17.9	35.8

**Table 2:** Content of polyphenols, flavonoids, phenolic acids, flavonols, and anthocyanins and chemical composition of colored potato pulp.

	**Marleta Blue**	**Magenta Love**
**Polyphenols**		
Total phenolic content—with F-C reagent (mg catechin/100 g d.m.)	188.01 ± 22.84 a	340.46 ± 35.18 b
Total phenolic content—without F-C reagent (mg catechin/100 g d.m.)	197.27 ± 7.19 a	366.54 ± 15.58 b
Flavonoids (mg rutin/100 g d.m.)	113.30 ± 18.53 a	148.75 ± 12.52 b
Phenolic acids (mg gallic acid/100 g d.m)	28.90 ± 0.00 a	61.16 ± 1.69 b
Flavonols (mg quercetin/100 g d.m.)	53.05 ± 0.00 b	8.54 ± 9.81 a
Anthocyanins (mg cyanidin—3-glucoside/100 g d.m.)	23.87 ± 0.92 a	49.27 ± 2.66 b
ABTS (mMTx/kg d.m.)	60.52 ± 0.70 a	77.31 ± 0.83 b
**Chemical composition (g/100 g d.m.)**		
Protein	4.35 ± 0.03 a	6.44± 0.11 b
Fat	0 ± 0	0 ± 0
Ash	2.01 ± 0 a	2.37 ± 0.07 b
Total dietary fiber	16.71 ± 0.31 a	19.68 ± 0.27 b

Different letters in the rows in the table represent the statistically significant difference of average values (*p* = 0.05).

**Table 3:** Content of polyphenols, flavonoids, anthocyanins, and phenolic acids in dessert cookies with the addition of colored potato pulp.

	TPC—with F-C Reagent (mg Catechin/100 g d.m.)	TTPC—without F-C Reagent (mg Catechin/100 g d.m.)	Flavonoids (mg Rutin/100 g d.m.)	Anthocyanins (mg Cyanidin—3-Glucoside/100 g d.m.)	Phenolic Acids (mg Gallic Acid/100g d.m.	ABTS (mMTx/kg d.m.)
Control	22.07 ± 4.14 a	18.22 ± 7.33 ab	14.40 ± 2.97 a	0.95 ± 1.06 a	2.94 ± 0.15 c	15.78 ± 0.85 a
DCMB5	30.46 ± 2.19 b	26.86 ± 3.45 b	17.75 ± 1.77 b	2.60 ± 0.00 b	1.87 ± 0.07 a	22.17 ± 0.10 b
DCMB10	33.74 ± 1.09 b	24.27 ± 1.73 b	23.5 ± 1.77 c	2.60 ± 0.00 b	1.99 ± 0 a	26.96 ± 0.10 c
DCML5	33.01 ± 1.26 b	26.50 ± 0.00 b	36.35 ± 3.61 d	1.00 ± 0.00 a	2.17 ± 0.04 b	25.36 ± 1.05 c
DCML10	47.60 ± 1.67 c	42.56 ± 3.99 c	61.25 ± 2.26 e	4.20 ± 0.00 c	6.05 ± 0.17 d	38.15 ± 0 d

Different letters in the column represent the statistically significant difference of average values (*p* = 0.05).

**Table 4:** Chemical composition of dessert cookies with the addition of colored potato pulp (g/100 g) and their energy value (kcal/100 g).

	Moisture	Protein	Fat	Ash	Total Dietary Fiber	Available Carbohydrates	Energy
Control	3.12 ± 0.05 a	1.18 ± 0.02 b	12.83 ± 0.02 a	1.45 ± 0.02 a	0.49 ± 0.02 b	80.93 ± 1.50 d	444.89 ± 3.20 c
DCMB5	4.15 ± 0.02 b	1.11 ± 0 a	12.85 ± 0.03 a	1.43 ± 0.05 a	0.22± 0 a	80.24 ± 1.12 c	441.49 ± 3.13 c
DCMB10	5.33 ± 0.07 c	1.12 ± 0.01 a	12.84 ± 0.05 a	1.47 ± 0.07 a	0.80 ± 0.07 c	78.44 ± 2.40 a	435.40 ± 2.90 a
DCML5	4.54 ± 0.03 b	1.19 ± 0.04 b	12.82 ± 0.03 a	1.44 ±0.09 a	0.54 ± 0.05 b	79.47 ± 2.12 b	439.10 ± 2.76 b
DCML10	5.10 ± 0.05 c	1.39 ± 0.05 c	12.78 ± 0.04 a	1.50 ± 0.01 a	1.19 ±0.04 d	78.04 ± 1.83 a	435.12 ± 2.55 a

Different letters in the column represent the statistically significant difference of average values (*p* = 0.05).

**Table 5:** Acrylamide and HMF in dessert cookies with the addition of colored potato pulp.

	Acrylamide Content (μg/kg)	HMF Content (μg/kg)
Control	65.67 ± 3.25 a	3.1 ± 0.1 b
DCMB5	80.50 ± 7.5 b	2.00 ± 0.008 a
DCMB10	90.11 ± 7.98 d	1.99 ± 0.02 a
DCML5	89.79 ± 1.64 b	1.96 ± 0.1 a
DCML10	95.11 ± 3.44 d	2.22 ± 0.18 a

Different letters in the column represent the statistically significant difference of average values (*p* = 0.05).

**Table 6:** Physical properties of dessert cookies enriched with pulp of colored potato varieties.

	Weight (g)	Volume (mL)	Diameter (mm)	Thickness (mm)	Spread Ratio
Control	10.15 ± 0.15 b	56.70 ± 0 a	61.58 ± 0.12 d	11.0 ± 0.10 a	5.59 ± 0.12 c
DCMB5	9.93 ± 0.10 a	58.80 ± 0.15 b	59.16 ± 0.37 c	11.3 ± 0.59 b	4.97 ± 0.20 b
DCMB10	10.18 ±0.20 b	61.00 ± 0.27 c	57.58 ± 0.50 b	12.00 ± 0.12 c	4.80 ± 0.20 b
DCML5	10.27 ± 0.10 b	61.30 ± 0.53 c	59.00 ± 0.20 c	12.5 ± 0.70 c	4.72 ± 0.43 b
DCML10	10.57 ± 0 c	58.00 ± 0.89 ab	56.58 ± 0 a	13.37 ± 0 d	4.23 ± 0.12 a

Different letters in the column represent the statistically significant difference of average values (*p* = 0.05).

## Data Availability

The data used to support the findings of this study can be made available by the corresponding author upon request.

## Supplementary Materials

The following supporting information can be downloaded at: https://www.mdpi.com/article/10.3390/foods12203735/s1, Figure S1: Measuring the volume of a biscuit using a Volscan Profiler laser volume analyzer (Stable Micro Systems, Godalming, UK).

## References

[1] FAO (2019). The State of Food and Agriculture 2019. Moving Forward on Food Loss and Waste Reduction.

[2] Sadh P.K., Duhan S., Duhan J.S. (2018). Agro-industrial Wastes and Their Utilization Using Solid State Fermentation: A Review. Bioresour. Bioproc..

[3] FAOSTAT (2021). Food and Agriculture Organization of the United Nations Statistical Database.

[4] Lemes A.C., Egea M.B., Oliveira Filho J.G., Gautério G.V., Ribeiro B.D., Coelho M.A.Z. (2022). Biological Approaches for Extraction of Bioactive Compounds from Agroindustrial By-products: A Review. Front. Bioeng. Biotechnol..

[5] Reguengo L.M., Salgaço M.K., Sivieri K., Maróstica M.R.J. (2022). Agro-industrial by-products: Valuable sources of bioactive compounds. Food Res. Int..

[6] Bartek L., Sundin N., Strid I., Andersson M., Hansson P.A., Eriksson M. (2022). Environmental benefits of circular food systems: The case of upcycled protein recovered using genome edited potato. J. Clean. Prod..

[7] Ismail S.A., Abdullah V.S., Kamel F.H. (2022). Extraction of α-solanine and α-chaconine from green potato tubers and evaluation of its antimicrobial activity. Plant Arch..

[8] Sip A., Thanh-Blicharz L., Siergiej K., Lesiecki M., Lewandowicz G. (2016). Odpady przemysłu ziemniaczanego jako podłoże do hodowli bakterii mlekowych. Postępy Nauk. Technol. Przemysłu Rolno-Spożywczego.

[9] Szarek D., Przewodowska A. (2016). Fizykochemiczne metody odzyskiwania białek z soku ziemniaka. Ziemn. Pol..

[10] Markiewicz M., Przewodowska A., Przewodowski W., Stochła W. (2015). Wykorzystanie chromatografii membranowej do odzyskiwania białek aktywnych biologicznie z odpadów przemysłu skrobiowego. Rocz. Ochr. Sr..

[11] Allied Market Research Report Confectionery Market by Product Type (Hard-Boiled Sweets, Mints, Gums & Jellies, Chocolate, Caramels & Toffees, Medicated Confectionery, Fine Bakery Wares, and Others), Age Group (Children, Adult, and Geriatric), Price Point (Economy, Mid-Range, and Luxury), and Distribution Channel (Supermarket/Hypermarket, Convenience Stores, Pharmaceutical & Drug Stores, Food Services, Duty-Free Outlets, E-Commerce, and Others): Global Opportunity Analysis and Industry Forecast, 2021–2027. https://www.alliedmarketresearch.com/confectionery-market.

[12] Stojiljković V., Todorović A., Pejić S., Kasapović J., Saičić Z.S., Radlović N., Pajović S.B. (2009). Antioxidant Status and LipidPeroxidation in Small Intestinal Mucosa of Children with Celiac Disease. Clin. Biochem..

[13] Caio G., Volta U., Sapone A., Leffler D.A., De Giorgio R., Catassi C., Fasano A. (2019). Celiac disease: A comprehensive current—Review. BMC Med..

[14] Filipčev B., Lević L., Bodroža-Solarov M., Mišljenović N., Koprivica G. (2010). Quality Characteristics and Antioxidant Properties of Breads Supplemented with Sugar Beet Molasses-Based Ingredients. Int. J. Food Prop..

[15] Altunkaya A., Hedegaard R.V., Brimer L., Gökmen V., Skibsted L.H. (2013). Antioxidant capacity versus chemical safety of wheat bread enriched with pomegranate peel powder. Food Funct..

[16] Świeca M., Gawlik-Dziki U., Dziki D., Baraniak B., Czyż J. (2013). The influence of protein-flavonoid interactions on protein digestibility in vitro and the antioxidant quality of breads enriched with onion skin. Food Chem..

[17] Korus A., Gumul D., Krystyjan M., Juszczak L., Korus J. (2017). Evaluation of the quality, nutritional value and antioxidant activity of gluten-free biscuits made from corn-acorn flour or corn-hemp flour composites. Eur. Food Res. Technol..

[18] Zhu F., Sun J. (2019). Physicochemical and sensory properties of steamed bread fortified with purple sweet potato flour. Food Biosci..

[19] Martirosyan D., Kanya H., Nadalet C. (2021). Can functional foods reduce the risk of disease? Advancement of functional food definition and steps to create functional food products. Funct. Food Health Dis..

[20] Wischmann B., Ahmt T., Bandsholm O., Blennow A., Young N., Jeppesen L., Thomsen L. (2007). Testing properties of potato starch from different scales of isolations—A ringtest. J. Food Eng..

[21] AOAC (2006). Official Methods of Analysis.

[22] REGULATION (EU) No 1169/2011 OF THE EUROPEAN PARLIAMENT AND OF THE COUNCILof 25 October 2011 on the Provision of Food Information to Consumers, Amending Regulations (EC) No 1924/2006 and EC) No 1925/2006 of the European Parliament and of the Council, and Repealing Commission Directive 87/250/EEC, Council Directive 90/496/EEC, Commission Directive 1999/10/EC, Directive 2000/13/EC of the European Parliament and of the Council, Commission Directives 2002/67/EC and 2008/5/EC and Commission Regulation (EC) No 608/2004. https://eur-lex.europa.eu/LexUriServ/LexUriServ.do?uri=OJ:L:2011:304:0018:0063:en:PDF.

[23] AACC (2000). Approved Methods of the American Association of Cereal Chemists.

[24] Singleton V.L., Orthofer R., Lamuela-Raventós R.M. (1999). Analysis of total phenols and other oxidation substrates and antioxidants by means of Folin-Ciocalteu reagent. Methods in Enzymology.

[25] El Hariri B., Sallé G., Andary C. (1991). Involvement of flavonoids in the resistance of two poplar cultivars to mistletoe (*Viscum album* L.). Protoplasma.

[26] Mazza G., Fukumoto L., Delaquis P., Girard B., Ewert B. (1999). Anthocyanins, phenolics, and color of Cabernet franc, Merlot, and Pinot noir wines from British Columbia. J. Agric. Food Chem..

[27] Oomah B.D., Cardador-Martínez A., Loarca-Piña G. (2005). Phenolics and antioxidative activities in common beans (*Phaseolus vulgaris* L.). J. Sci. Food Agric..

[28] Re R., Pellegrini N., Proteggente A., Pannala A., Yang M., Rice-Evans C. (1999). Antioxidant activity applying an improved ABTS radical cation decolorization assay. Free Radic. Biol. Med..

[29] Surma M., Sadowska-Rociek A., Cieślik E., Sznajder-Katarzyńska K. (2017). Optimization of QuEChERS sample preparation method for acrylamide level determination in coffee and coffee substitutes. Microchem. J..

[30] Marconi O., Bravi E., Perretti G., Martini R., Montanari L., Fantozzi P. (2010). Acrylamide risk in food products: The shortbread case study. Anal. Methods.

[31] Lachman J., Hamouz K., Šulc M., Orsák M., Pivec V., Hejtmánková A., Dvořák P., Čepl J. (2009). Cultivar differences of total anthocyanins and anthocyanidins in red and purple-fleshed potatoes and their relation to antioxidant activity. Food Chem..

[32] Navarre D.A., Pillai S.S., Shakya R., Holden M.J. (2011). HPLC profiling of phenolics in diverse potato genotypes. Food Chem..

[33] Kita A., Bąkowska-Barczak A., Lisińska G., Hamouz K., Kułakowska K. (2015). Antioxidant activity and quality of red and purple flesh potato chips. LWT—Food Sci. Technol..

[34] Middleton E., Kandaswami C., Theoharides T.C. (2000). The effects of plant flavonoids on mammalian cells: Implications for inflammation, heart disease, and cancer. Pharmacol. Rev..

[35] Manach C., Mazur A., Scalbert A. (2005). Polyphenols and prevention of cardiovascular diseases. Curr. Opin. Lipidol..

[36] Ramos S. (2007). Effects of dietary flavonoids on apoptotic pathways related to cancer chemoprevention. J. Nutr. Biochem..

[37] Yokohira M., Yamakawa K., Saoo K., Matsuda Y., Hosokawa K., Hashimoto N., Kuno T., Imaida K. (2008). Antioxidant effects of flavonoids used as food additives (purple corn color, enzymatically modified isoquercitrin, and isoquercitrin) on liver carcinogenesis in a rat medium-term bioassay. J. Food Sci..

[38] Balasuriya N., Rupasinghe H.V. (2012). Antihypertensive properties of flavonoid-rich apple peel extract. Food Chem..

[39] Makarova E., Górnaś P., Konrade I., Tirzite D., Cirule H., Gulbe A., Pugajeva I., Seglina D., Dambrova M. (2014). Acute anti-hyperglycaemic effects of an unripe apple preparation containing phlorizin in healthy volunteers: A preliminary study. J. Sci. Food Agric..

[40] Everette J.D., Bryant Q.M., Green A.M., Abbey Y.A., Wangila G.W., Walker R.B. (2010). Thorough study of reactivity of various compound classes toward the Folin−Ciocalteu reagent. J. Agric. Food Chem..

[41] Gulcin İ. (2020). Antioxidants and antioxidant methods: An updated overview. Arch. Toxicol..

[42] Korus J., Juszczak L., Ziobro R., Witczak M., Grzelak K., Sójka M. (2012). Defatted strawberry and blackcurrant seeds as functional ingredients of gluten-free bread. J. Texture Stud..

[43] Gumul D., Ziobro R., Korus J., Kruczek M. (2021). Apple pomace as a source of bioactive polyphenol compounds in gluten-free breads. Antioxidants.

[44] Sudha M., Baskaran V., Leelavathi K. (2007). Apple pomace as a source of dietary fiber and polyphenols and its effect on the rheological characteristics and cake making. Food Chem..

[45] Ajila C., Leelavathi K., Rao U.P. (2008). Improvement of dietary fiber content and antioxidant properties in soft dough biscuits with the incorporation of mango peel powder. J. Cereal Sci..

[46] Bertagnolli S.M.M., Silveira M.L.R., de Oliveira Fogaça A., Umann L., Penna N.G. (2014). Bioactive compounds and acceptance of cookies made with Guava peel flour. Food Sci. Technol..

[47] Kopeć A., Syska A., Leszczyńska T., Piątkowska E. (2014). Skład chemiczny i aktywność antyoksydacyjna biszkoptów i ciastek bezglutenowych. Właściwości Produktów Surowców Żywnościowych. Wybrane Zagadnienia.

[48] Aboshora W., Yu J., Omar K.A., Li Y., Hassanin H.A.M., Navicha W.B., Zhang L. (2019). Preparation of Doum fruit (*Hyphaene thebaica*) dietary fiber supplemented biscuits: Influence on dough characteristics, biscuits quality, nutritional profile and antioxidant properties. J. Food Sci. Technol..

[49] Maner S., Sharma A.K., Banerjee K. (2015). Wheat flour replacement by wine grape pomace powder positively affects physical, functional and sensory properties of cookies. Proc. Natl. Acad. Sci. India Sect. B Biol. Sci..

[50] Ajibola C., Oyerinde V., Adeniyan O. (2015). Physicochemical and antioxidant properties of whole-wheat biscuits incorporated with Moringa oleifera leaves and cocoa powder. J. Sci. Res. Rep..

[51] Hodas F., Rosa M., Zorzenon T., Milani P.G. (2021). Moringa oleifera potential as afunctional food and a natural food additive: A biochemical approach. Ann. Acad. Bras. Cienc..

[52] Natukunda S., Muyonga J.H., Mukisa I.M. (2015). Effect of tamarind (*Tamarindus indica* L.) seed on antioxidant activity, phytocompounds, physicochemical characteristics, and sensory acceptability of enriched cookies and mango juice. Food Sci. Nutr..

[53] Klunklin W., Savage G. (2018). Effect of substituting purple rice flour for wheat flour on physicochemical characteristics, in vitro digestibility, and sensory evaluation of biscuits. J. Food Qual..

[54] Van Toan N., Anh V.Q. (2018). Preparation and improved quality production of flour and the made biscuits from purple sweet potato. J. Food Nutr..

[55] Pasqualone A., Makhlouf F.Z., Barkat M., Difonzo G., Summo C., Squeo G., Caponio F. (2019). Effect of acorn flour on the physico-chemical and sensory properties of biscuits. Heliyon.

[56] Ścibisz I., Kalisz S., Mitek M. (2010). Termiczna degradacja antocyjanow owocow borowki wysokiej. Zywnosc. Nauka. Technologia. Jakosc.

[57] COMMISSION REGULATION (EU) 2017/2158 of 20 November 2017 Establishing Mitigation Measures and Benchmark Levels for the Reduction of the Presence of Acrylamide in Food. https://op.europa.eu/en/publication-detail/-/publication/1f3b45fb-ce6b-11e7-a5d5-01aa75ed71a1/language-en.

[58] Saturni L., Ferretti G., Bacchetti T. (2010). The Gluten-Free Diet: Safety and Nutritional Quality. Nutrients.

[59] Hopper A.D., Hadjivassiliou M., Butt S., Sanders D.S. (2007). Adult Coeliac Disease. BMJ.

[60] Sun L., Miao M. (2020). Dietary polyphenols modulate starch digestion and glycaemic level: A review. Crit. Rev. Food Sci. Nutr..

[61] Hřivna L., Zigmundová V., Burešová I., Maco R., Vyhnánek T., Trojan V. (2018). Rheological properties of dough and baking quality of products using coloured wheat. Plant Soil Environ..

[62] Liu N., Ma S., Wang Z., Li L., Zheng X., Wang X. (2020). Influence of wheat bran dietary fiber on gluten protein structure during dough fermentation. J. Food Process. Preserv..

[63] Wyrwisz J., Półtorak A., Moczkowska M., Żontała K., Stelmasiak A., Łopacka J., Ulanicka U., Wierzbicka A. (2013). Wpływ stopnia rozdrobnienia preparatów wysokobłonnikowych na właściwości reologiczne ciasta pszennego. Inżynieria Rol..

[64] Yadav R.B., Yadav B.S., Dhull N. (2011). Effect of incorporation of plantain and chickpea flours on the quality characteristics of biscuits. J. Food Sci. Technol..

[65] Awolu O.O., Sudha L.M., Manohar B. (2018). Influence of defatted mango kernel seed flour addition on the rheological characteristics and cookie making quality of wheat flour. Food Sci. Nutr..

[66] Tavares B.O., Silva E.P.D., Silva V.S.N.D., JÚnior M.S.S., Ida E.I., Damiani C. (2016). Stability of gluten free sweet biscuit elaborated with rice bran, broken rice and okara. Food Sci. Technol..

[67] Parveen H., Bajpai A., Bhatia S., Singh S. (2017). Analysis of biscuits enriched with fibre by incorporating carrot and beetroot pomace powder. Indian J. Nutr. Diet..

[68] Srivastava S., Singh K. (2016). Physical, sensory and nutritional evaluation of biscuits prepared by using beetroot (Beta vulgaris) powder. Int. J. Innov. Res. Adv. Stud..

[69] Jothi J., Hashem S., Rana M., Rahman M., Shams-Ud-Din M. (2014). Effect of gluten-free composite flour on physico-chemical and sensory properties of cracker biscuits. J. Sci. Res..

[70] Makinde F.M., Taibat A.A. (2018). Quality characteristics of biscuits produced from composite flours of wheat, corn, almondand coconut. Ann. Food Sci. Technol..

[71] Adeola A.A., Ohizua E.R. (2018). Physical, chemical, and sensory properties of biscuits prepared from flour blends of unripe cooking banana, pigeon pea, and sweet potato. Food Sci. Nutr..

