# Supplementary material for: Pulp from Colored Potatoes (Solanum tuberosum L.) as an Ingredient Enriching Dessert Cookies
Source: Foods. 2023 Oct 11;12(20):3735. doi: 10.3390/foods12203735 (PMC10606129; doi:10.3390/foods12203735)
Supplement: Supplementary file 1 [file foods-12-03735-s001.zip › foods-2656707-supplementary.pdf]

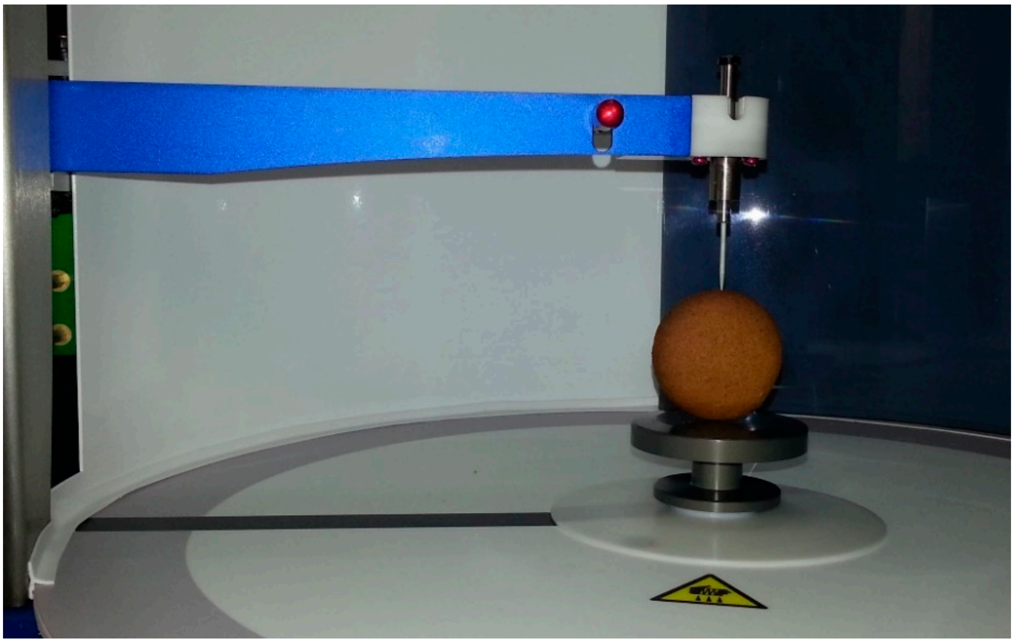

Sf1.

Measuring the volume of a biscuit using a Volscan Profiler laser volume analyzer (Stable Micro Systems, England)
